# Beyond Trade-Offs: Autonomy, Effectiveness, Fairness, and Normativity in Risk and Crisis Communication

**DOI:** 10.1080/15265161.2024.2353826

**Published:** 2024-05-20

**Authors:** Federico Germani, Giovanni Spitale, Nikola Biller-Andorno

**Affiliations:** Institute of Biomedical Ethics and History of Medicine (IBME), University of Zurich

**Keywords:** Public health ethics, citizen engagement, democratic technologies, risk and crisis communication

## Abstract

This paper addresses the critiques based on trade-offs and normativity presented in response to our target article proposing the Public Health Emergency Risk and Crisis Communication (PHERCC) framework. These critiques highlight the ethical dilemmas in crisis communication, particularly the balance between promoting public autonomy through transparent information and the potential stigmatization of specific population groups, as illustrated by the discussion of the mpox outbreak among men who have sex with men. This critique underscores the inherent tension between communication effectiveness and autonomy versus fairness and equity. In response, our paper reiterates the adaptability of the PHERCC framework, emphasizing its capacity to tailor messages to diverse audiences, thereby reducing potential stigmatization and misinformation. Through community engagement and feedback integration, the PHERCC framework aims to optimize the effectiveness of communication strategies while addressing ethical concerns. Furthermore, by involving affected communities in the communication strategy from the onset, the framework seeks to minimize ethical trade-offs and enhance the acceptance and effectiveness of public health messages.

In response to our proposal for the PHERCC (Public Health Emergency Risk and Crisis Communication) framework (Spitale, Germani, and Biller-Andorno 2024), Bernstein et al. offered a critique, emphasizing the necessity for ethical Risk and Crisis Communication (RCC) to acknowledge the existence of tradeoffs (Bernstein, Barnhill, and Faden 2024). Specifically, they highlighted the delicate balance between providing accurate information to the public to promote autonomy and the potential risks associated with such information, such as stigma. In their own words:

[…] consider a communicable disease that disproportionately affects certain groups in a population. Communicating to the public that this disease primarily affects these groups could respect autonomy or promote overall well-being. At the same time, however, emphasizing that only some groups are at serious risk could also expose members of those groups to stigma and disdain, especially if the affected groups are already subject to discrimination or unfair disadvantage. (Bernstein, Barnhill, and Faden 2024)

To substantiate their claim, they draw upon an example from the recent monkeypox outbreak (2022-2023). They illustrate this point by stating:

A recent example is the mpox outbreak […], in which most reported cases were among men who have sex with men. Consider a government’s decision to clearly state, as part of its communication with the public about the mpox outbreak, that men who have sex with men are at higher risk of being exposed to mpox than the general population. Such communication provides information to men who have sex with men, and this information might help them to protect themselves (for example, by getting vaccinated against mpox virus). But such communication also risks stigmatizing men who have sex with men. This stigma—and the discrimination that may accompany it—are forms of group-based inequity. (Bernstein, Barnhill, and Faden 2024)

Bernstein et al. contend that there is a tension, “[…] *between effectiveness and autonomy on the one hand, and fairness as equity on the other. […] they must recognize that this tension exists.”* (Bernstein, Barnhill, and Faden 2024)

In line with previous considerations, in particular raised in the context of mpox (März, Holm, and Biller-Andorno 2022; World Health Organization 2023), Bernstein et al. raise valid concerns regarding the potential tradeoffs between effectiveness and autonomy on the one hand, and fairness and equity on the other hand. However, our PHERCC framework acknowledges that communication is not a one-size-fits-all approach. It acknowledges the importance of targeting specific communities with tailored messages, aligning with the evolving consensus in infodemic management, including public health communication and RCC. A targeted approach, as outlined in our PHERCC framework, can mitigate the risk of stigma by ensuring that communication resonates with the intended audiences (i.e. *publics* in our PHERCC framework) and addresses their unique needs, vulnerabilities, and understanding of information (Spitale, Germani, and Biller-Andorno 2024). Simultaneously, it minimizes the dissemination of unnecessary information to audiences that do not require it for their health, providing them with adapted messages that cater to their specific needs. In the context of the mpox example, this could involve delivering different messages to gay communities and the broader public through varied channels. For instance, gay communities could be informed about the transmission risks associated with specific sexual behaviors, while non-gay communities could be informed that monkeypox transmission is not confined to gay communities, emphasizing that transmission is linked to sexual activity rather than sexual orientation. Furthermore, by involving communities in the strategy definition and communication design process, the PHERCC framework ensures that their perspectives are incorporated from the outset, minimizing potential harms such as stigma. The empirical approach (involving community engagement and feedback-loop integration) in our PHERCC framework underscores a commitment to maximizing the effectiveness of communication strategies while mitigating ethical concerns. While a tradeoff between autonomy/effectiveness and fairness/equity exists, as pointed out by Bernstein et al., the utilization of the PHERCC framework should allow for its significant minimization.

The authors provide another example, stating that “[…] *older individuals and people with various comorbidities are at especially high risk of becoming seriously ill from a COVID-19 infection. Here, again, emphasizing the elevated risk of these groups in public health communications is ethically fraught precisely because ethical values come into tension. At the height of the pandemic, groups at elevated risk faced stigma as well as resentment from low-risk groups, including calls for increased isolation of the higher-risk groups to preserve liberties for low-risk groups. Emphasizing the higher risks of some groups may have led individuals to take fewer precautions and thereby (indirectly) impose greater risk on at-risk individuals. This is not to say that PHERCC should have omitted this information, but rather to highlight that PHERCC will often involve tradeoffs between different values.” (*Bernstein, Barnhill, and Faden 2024*)*

In this passage, the authors raise a concern regarding the potential consequences of emphasizing the higher risks faced by particularly vulnerable groups, such as older individuals during the COVID-19 pandemic or those with underlying health conditions. They highlight two main issues: a) Stigma toward these populations may arise due to the highlighting of their elevated risk, and b) individuals with lower risk may perceive themselves as less susceptible and consequently take fewer precautions, inadvertently increasing the risk for at-risk individuals.

Similar to our previous point, it is crucial to acknowledge that communication strategies cannot be universally applied. The needs and vulnerabilities of different demographic groups vary significantly, highlighting the necessity for tailored communication approaches (Hyland-Wood et al. 2021; Rämgård et al. 2023). In the context of the PHERCC framework, this means acknowledging that communication for low-risk and high-risk groups will inherently differ. For example, for low-risk groups, communication may focus on the importance of solidarity and collective responsibility, emphasizing the role each individual plays in protecting vulnerable members of society. Conversely, communication directed toward high-risk groups should prioritize providing clear and actionable guidance to mitigate their risk. Once again, the overarching aim of the PHERCC framework is to adopt an inclusive and empirical approach. This involves actively engaging with affected communities in the communication process and considering their perspectives, thereby ensuring both the effectiveness of public health messaging and the minimization of harm.

Kabasenche also raises a similar concern regarding tradeoffs between autonomy and fairness:

Not all of us will be as optimistic as they are that ethical judgment in a public health emergency involves no trade-offs. […] At the least, we should all be clear about why we make trade-offs as we do, and we should subject our judgment to scrutiny from others. This is true of any citizens weighing in, as well as for public health decision makers. (Kabasenche 2024*)*

Regarding Kabasenche’s assertion that we believe PHERCC actions should involve no tradeoffs between fairness and autonomy/effectiveness, this is a misinterpretation. Our point is that while tradeoffs exist, as discussed above, PHERCC practices should strive to minimize them by ensuring that actions are perceived as fair by the public. As previously noted in response to Bernstein et al.’s concerns, the key aspect in minimizing the impact and extent of such tradeoffs lies in how we define the *effectiveness* of communication. Effective communication, as elucidated by our PHERCC framework, necessitates proper design elements such as the selection of the target audience for specific messages, utilization of appropriate communication channels, thoughtful messaging choices, and most importantly, the integration of the public in the design of communication strategies and campaigns. By prioritizing effectiveness through these design principles, we work toward minimizing ethical tradeoffs between autonomy and fairness. In other words, striving for effectiveness is not in conflict *with* ensuring the respect of ethical considerations and principles; rather, effectiveness can be pursued *by* ensuring the respect of ethical consideration and principles.

Kabasenche brings forth concerns about the tradeoffs between autonomy and fairness within the context of normativity, contending that our PHERCC framework is inaccurate due to its lack of consideration of normativity (Kabasenche 2024). The author states that:

One immediate concern with this interpretation is that I am not sure the authors actually agree with it themselves, despite their seeming to endorse it at points. They do seem to believe masks should be worn in certain settings (imagine a sign outside a hospital at the height of a surge that says “Here’s some information, but you can choose whether to wear a mask or not. Or, if you’d like, tell us why you are not wearing one”). If that is their view, they should be transparent about the ethical considerations that lead them to that judgment, as should any public health decision makers. […] But if Spitale et al. truly do believe PHERCC action should only be information-dispensing and not involve normative policy, then they ought not believe it. Public health emergencies almost inevitably require coordinated action. Spitale et al. seem dangerously close to endorsing a kind of reverse Humeanism—believing that a shared judgment about what we ought to do simply will come about as a result of really accurate PHERCC. (Kabasenche 2024)

The concerns raised by the author appear to misconstrue our stance on the role of PHERCC actions in public health emergencies. Firstly, it is important to clarify that our position does not advocate for a solely information-dispensing approach. Rather, we emphasize the importance of transparent and ethical decision-making processes that involve both information dissemination and normative policy considerations. Furthermore, the author’s concern about basing decisions on non-normativity leading to individuals entering hospitals without masks overlooks the separation between communication and normative policy. While communication of normative aspects follows the principles of the PHERCC framework, the actual implementation of policies, such as mask mandates, is a separate issue—and was not the focus of our original paper, which as the title itself states, concerns Risk and Crisis Communication. Although not explicitly articulated in our PHERCC framework, improving understanding through effective communication can lead to better behavioral outcomes (Heydari et al. 2021; Porat et al. 2020). When individuals comprehend the rationale (facilitated by communication) behind certain measures, such as wearing masks in hospitals, they are more inclined to voluntarily comply (Anderson and Hobolt 2022). Consequently, when such measures are eventually mandated, there is likely to be greater adherence, thereby diminishing the necessity for extensive communication efforts to explain the importance of, for instance, mask mandates in safeguarding public health. Again, it is crucial to stress that this does not imply that mask or vaccine mandates should not be imposed, nor has this been explicitly or implicitly considered in our PHERCC framework. In fact, pockets of resistance against measures to safeguard public health would likely persist, irrespective of the flawless functioning of PHERCC. However, communication plays a significant role, and if it can help reduce the percentage of people opposing health-protective behaviors, then policies aimed at safeguarding public health could obtain stronger support from a larger segment of the population.
